# Perimenopausal Effects of Estradiol on Anhedonia and Psychosis Study (PEEPs): study protocol for a neural and molecular mechanistic clinical trial

**DOI:** 10.1186/s13063-023-07166-7

**Published:** 2023-02-28

**Authors:** Melissa J. M. Walsh, Kathryn Gibson, Megan Hynd, Tory A. Eisenlohr-Moul, Erin C. Walsh, Lauren Schiff, Fred Jarskog, David Lalush, Gabriel S. Dichter, Crystal E. Schiller

**Affiliations:** 1 Carolina Institute for Developmental Disabilities, University of North Carolina at Chapel Hill School of Medicine, Chapel Hill, NC 27510 USA; 2 Department of Psychiatry, University of North Carolina at Chapel Hill, Chapel Hill, NC 27514 USA; 3 Department of Psychology and Neuroscience, University of North Carolina at Chapel Hill, Chapel Hill, NC 27514 USA; 4 Department of Psychiatry, University of Illinois at Chicago, Chicago, IL USA; 5 Department of Obstetrics and Gynecology, University of North Carolina at Chapel Hill, Chapel Hill, NC USA; 6 North Carolina Psychiatric Research Center, Raleigh, NC 27610 USA; 7 Joint Department of Biomedical Engineering, University of North Carolina at Chapel Hill and North Carolina State University, Raleigh, NC USA

**Keywords:** Menopause, Depression, Anhedonia, Psychosis, Estrogen, Rewards, fMRI, PET, Mechanistic trial

## Abstract

**Background:**

The perimenopausal transition is accompanied by psychiatric symptoms in over 10% of women. Symptoms commonly include depressed mood and anhedonia and less commonly include psychosis. Psychiatric symptoms have been linked to the depletion and/or variability of circulating estradiol, and estradiol treatment reduces perimenopausal anhedonia and psychosis in some women. Estrogen fluctuations may disrupt function in the mesolimbic reward system in some women, leading to psychiatric symptoms like anhedonia or psychosis. The Perimenopausal Effects of Estradiol on Anhedonia and Psychosis Study (PEEPs) is a mechanistic clinical trial that aims to (1) identify relationships between perimenopausal-onset anhedonia and psychosis and neuromolecular markers of mesolimbic reward responses and (2) determine the extent to which estradiol treatment-induced changes in mesolimbic reward responses are associated with alleviation of perimenopausal onset anhedonia or psychosis.

**Methods:**

This study will recruit 100 unmedicated women ages 44–55 in the late-stage perimenopausal transition, sampling across the range of mild-to-high anhedonia and absent-to-moderate psychosis symptoms. Patients will be randomized to receive either estradiol or placebo treatment for 3 weeks. Clinical outcome measures will include symptoms of anhedonia (measured with Snaith–Hamilton Pleasure Scale; SHAPS) and psychosis (measured with Brief Psychiatric Rating Scale; BPRS psychosis subscale) as well as neural markers of mesolimbic reward system functioning, including reward-related fMRI activation and PET-derived measure of striatal dopamine binding. Pre-treatment associations between (1) SHAPS/BPRS scores and (2) reward-related striatal dopamine binding/BOLD activation will be examined. Furthermore, longitudinal mixed models will be used to estimate (1) symptom and neuromolecular trajectories as a function of estradiol vs. placebo treatment and (2) how changes in reward-related striatal dopamine binding and BOLD activation predict variability in symptom trajectories in response to estradiol treatment.

**Discussion:**

This clinical trial will be the first to characterize neural and molecular mechanisms by which estradiol treatment ameliorates anhedonia and psychosis symptoms during the perimenopausal transition, thus laying the groundwork for future biomarker research to predict susceptibility and prognosis and develop targeted treatments for perimenopausal psychiatric symptoms. Furthermore, in alignment with the National Institute for Mental Health Research Domain Criteria initiative, this trial will improve our understanding of a range of disorders characterized by anhedonia, psychosis, and reward system dysfunction.

**Trial registration:**

ClinicalTrials.gov NCT05282277

## Administrative information

**Table:** 

Title {1}	Perimenopausal Effects of Estradiol on Anhedonia and Psychosis Study (PEEPs): Study Protocol for a Neural and Molecular Mechanistic Clinical Trial
Trial registration {2a}	ClinicalTrials.gov Identifier: NCT05282277
Trial registration: data set {2a}	n/a; the register used for registration does not collect all items from the World Health Organization Trial Registration Data Set.
Protocol version {3}	This is version 1.6 of the study protocol (06/09/2022).
Funding {4}	National Institute of Mental Health (R01 MH128238 to DL, CES, and GSD and K23 MH113733 to EW).
Author details {5a}	1) Carolina Institute for Developmental Disabilities, University of North Carolina at Chapel Hill School of Medicine, Chapel Hill, NC 27510, USA. *to whom correspondence should be addressed: melissa_walsh@med.unc.edu 2) Department of Psychology and Neuroscience, University of North Carolina at Chapel Hill, Chapel Hill, NC 27514, USA. 3) Department of Psychiatry, University of Illinois at Chicago, Chicago, IL, USA. 4) Department of Psychiatry, University of North Carolina at Chapel Hill, Chapel Hill, NC 27514, USA. 5) Department of Obstetrics and Gynecology, University of North Carolina at Chapel Hill, Chapel Hill, North Carolina. 6) North Carolina Psychiatric Research Center, Raleigh, NC, 27610, USA 7) Joint Department of Biomedical Engineering, University of North Carolina at Chapel Hill and North Carolina State University, Raleigh, NC, USA
Name and contact information for the trial sponsor {5b}	National Institute of Mental Health; nimhinfo@nih.gov
Role of sponsor {5c}	The National Institute of Mental Health has no role in study design; collection, management, analysis, and interpretation of data; writing of the report; and the decision to submit the report for publication, or any ultimate authority over any of these activities.

## Introduction

### Background and rationale {6a}

The lifetime prevalence of affective disorders is two times greater in women than men [1], and reproductive transitions and associated neuroendocrine changes open windows of psychiatric vulnerability [2]. During the menopause transition, women show an increased susceptibility to a range of psychiatric symptoms, including depressed mood and anhedonia [3, 4] and, less commonly, psychosis [5]. Estradiol fluctuations are associated with depressive symptoms [6] and predict heightened risk for perimenopausal affective disorders [7]. Furthermore, estradiol treatment has been shown to alleviate perimenopausal-onset depression [8–10] and psychosis [5, 11] symptoms in some but not all women. While estradiol changes during the perimenopause have been linked to affective and psychotic symptoms, not all women show susceptibility to perimenopausal-onset psychiatric symptoms and response to estradiol treatment is mixed. Thus, research is needed to characterize the neurobiological mediators of susceptibility and response to treatment during the perimenopause.

Preclinical models of menopause suggest changes in striatal dopaminergic neurotransmission may, in part, mediate the relationship between ovarian hormone changes and depressive-like behaviors [12]. However, such findings remain to be validated in clinical studies. The NIMH-funded Perimenopausal Effects of Estradiol on Anhedonia and Psychosis Study (PEEPs), submitted in response to RFA-MH-21-105, aims to characterize the neural and molecular mechanisms, specifically focusing on mesolimbic responses to rewards, by which perimenopausal estradiol treatment influences affective and psychotic symptoms. The overarching goals of this study are to characterize potential neurobiological mediators of perimenopausal-onset anhedonia and psychosis to deliver validated molecular imaging targets to use in future mechanistic clinical trials of novel treatments for perimenopausal-onset psychiatric disorders and to inform future research examining the neural and molecular effects of ovarian hormone fluctuations across female reproductive transitions.

Anhedonia is amongst the most common affective symptoms associated with the menopause transition [13] and is broadly associated with ovarian hormone withdrawal in women and preclinical models of menopause [14]. Whereas psychosis is less common during the menopause transition, late-onset psychotic disorders (e.g., age at onset > 40 years) are twice as prevalent in women [15], with peak psychosis risk around the average age of perimenopause [5]. Both preclinical and human studies have shown an association between greater psychosis-spectrum behaviors and lower estrogen levels in females [5].

Dysfunction in reward salience processing is thought to contribute to both anhedonia and psychosis. Anhedonia is characterized by blunted reactivity to rewards and reduced salience attribution to reward cues [16], processes that are mediated by the mesolimbic dopamine system [17]. Preclinical evidence highlights that dopamine blockade produces anhedonic responses to rewarding stimuli [18]. The magnitude of phasic dopamine release to rewards predicts motivation to work for rewards in humans [19], and those with depression show reduced striatal dopaminergic activity during reward processing [20, 21]. However, other PET studies of dopamine activity in depressed samples have shown mixed findings (e.g., reduced, increased, or similar activity compared to healthy controls), which may be due to heterogeneity of symptoms in samples with major depression [22].

In contrast to the reduced salience attribution to rewards seen in anhedonia, salience attribution to irrelevant stimuli may contribute to psychotic symptoms [16], mediated by aberrant striatal dopamine release [23]. PET imaging studies of psychosis have shown higher baseline dopamine synthesis capacity in psychosis [24] as well as higher spontaneous dopamine release [25]. While distinct profiles of reward system dysfunction contribute to anhedonia and psychosis, dysregulation of the mesolimbic dopamine system may be a common etiological pathway.

Given that estradiol modulates striatal reward responses [26], ovarian hormone fluctuations and eventual depletion during the menopause transition may trigger the onset of aberrant dopamine function and thereby produce anhedonia and/or psychosis symptoms. Similarly, estradiol administration may ameliorate perimenopausal anhedonia and/or psychosis symptoms via its effect on the mesolimbic dopamine system. Importantly, both anhedonia and psychosis are associated with significant functional impairment [27, 28]. The identification of neural and molecular biomarkers sensitive to changes in anhedonia and psychosis severity during perimenopause may be used as mechanistic endpoints in perimenopause clinical trials of novel treatments for these symptoms.

### Approach novelty

Consistent with the NIMH Research Domain Criteria (RDoC) framework, this mechanistic trial uses dimensional measures of anhedonia and psychosis severity to stratify participants and as clinical outcome measures. Studies of transdiagnostic and neurobiologically grounded symptom dimensions have the potential to increase the pace of discoveries related to the pathogenesis and treatment of psychiatric disorders relative to categorical and polythetic approaches that treat diverse illness presentations as equivalent [29, 30]. This study focuses on neural responses to rewards as indices of the RDoC Positive Valence System constructs of approach motivation and initial responsiveness to rewards, constructs related to anhedonia in preclinical models [22, 31] and patient samples [32, 33]. Although psychosis is most commonly subsumed within the RDoC domain of Cognitive Systems [34], the present trial focuses on linkages between psychosis severity and response to estradiol treatment and striatal responses to rewards given associations between psychosis, neural response to rewards, and DA release to irrelevant stimuli [24, 25].

A particular novelty of this trial design is its use of DSM-based exclusion criteria in complement with transdiagnostic inclusion criteria. Disorders that are associated with reward system dysfunction unrelated to reproductive transitions are considered exclusionary (e.g., attention deficit hyperactivity disorder; recent moderate-to-severe substance-use disorders; chronic history of depressive episodes). The intent of this approach is to increase sample homogeneity to specifically target anhedonia and psychosis resulting from reproductive transition-related reward system dysfunction.

### Objectives {7}

The primary endpoints of the PEEPs study are mechanistic, measured via neural and molecular reward responses. Using simultaneous PET-MR imaging, this study will examine striatal blood oxygen level-dependent (BOLD) activation to rewards, measured with fMRI, and reward-related striatal dopamine release, measured with [^11^C]raclopride PET. To elicit a reward response, participants will complete a monetary incentive delay (MID) task during imaging. This task has previously shown sensitivity to altered reward-related dopamine release in a transdiagnostic anhedonia sample [35]. The first study objective aims to characterize the relationship between striatal reward responses and perimenopausal-onset anhedonia and psychosis symptoms at baseline. We hypothesize that anhedonia symptoms will be linked to reduced striatal BOLD activation and decreased task-related dopamine release to rewards. In contrast, we expect psychosis symptoms will be linked to increased striatal activation to rewards and higher baseline (tonic) dopamine release. The second objective of this trial will be to determine the effects of estradiol versus a placebo on perimenopausal-onset anhedonia and psychosis symptoms as well as striatal reward responses. We expect the effects of estradiol will be greater than placebo, as indexed by alleviation of anhedonia and psychosis symptoms and normalization of striatal reward response. We further hypothesize that reductions in *anhedonia* symptoms in the estradiol treatment group will be linked to increased striatal activation and increased dopamine release to rewards, whereas reductions in *psychosis* symptoms will be associated with decreased striatal activation to rewards and reduced tonic dopamine release.

### Trial design {8}

A parallel-group, randomized, placebo-controlled, double-blind superiority trial will be used to determine the effects of 3 weeks of estradiol treatment, relative to placebo, on perimenopausal-onset anhedonia and/or psychosis symptoms and neural and molecular reward responses. The study will enroll 100 participants with at least clinically significant anhedonia, including a range of mild-to-moderate anhedonia and absent-to-moderate psychosis symptoms. Based on screen failures and dropout rates in our prior studies involving PET-MR and perimenopausal depression, we expect to obtain analyzable data from at least 85 participants with two PET-MR scans each.

## Methods: participants, interventions, and outcomes

### Study setting {9}

The study setting is the Department of Psychiatry in the UNC-Chapel Hill School of Medicine.

### Eligibility criteria {10}

The study will include unmedicated, perimenopausal outpatient women ages 44–55 who are in late perimenopause and experiencing anhedonia and/or psychosis symptoms with onset coinciding with perimenopausal menstrual irregularity. To determine participant eligibility, women will undergo initial online and phone screening, the structured clinical interview for the DSM-5 (SCID-5), laboratory testing, and a pelvic exam/PAP test (or a medical records review for an exam within 3 years). Participants must meet the following inclusion criteria to be enrolled in the study:

- 44–55 years old, unmedicated, naturally perimenopausal woman in stage −1 of the Stages of Reproductive Aging Workshop (STRAW) criteria [36, 37]
- At least mild perimenopausal-onset anhedonia (SHAPS≥20) (Snaith et al. [38])
- Absent-to-moderate perimenopausal-onset psychosis spectrum symptoms as measured by the Brief Psychiatric Rating Scale (BPRS-Positive Symptom Subscale Score between 6 and 17) (Hafkenscheid [39]; Overall & Gorham [40])

Dimensional measures of anhedonia and psychosis severity are used to stratify participants. With respect to stratification, equal numbers of participants will be recruited into each of the following four strata:

1. Mild-to-moderate anhedonia + absent-to-mild psychosis
2. Mild-to-moderate anhedonia + moderate psychosis
3. High anhedonia + absent-to-mild psychosis
4. High anhedonia + moderate psychosis

Mild–moderate anhedonia severity is defined in this trial as Snaith–Hamilton Pleasure Scale (SHAPS [38]) scores between 20 and 36, and high anhedonia severity is defined as SHAPS>36 (using the ordinal scoring of Franken et al. [41]). There is no widely accepted clinical cut-off on the SHAPS, though a meta-analysis by Trøstheim and colleagues (2020) [42] reported that caseness is indicated by scoring more than 1.96 SDs above the general population, which would correspond to a SHAPS score of 25 or greater (see also Alsayednasser and colleagues [43]). However, in this trial, a SHAPS score cutoff of >20 was used to indicate at least mild, clinically impairing anhedonia, consistent with the cutoff used in the NIMH Fast-Fail Trial for Mood and Anxiety Disorders [38, 44]. The SHAPS has shown adequate psychometric properties in clinical and non-clinical samples [45], and ROC analyses suggest that a SHAPS score of ≥ 20 corresponds to clinically significant anhedonia from a general population sample [38].

Absent-to-mild psychosis severity is defined in this trial as <6 on the Brief Psychiatric Rating Scale (BPRS) positive symptom subscale [39, 40], consisting of four items: conceptual disorganization, suspiciousness, hallucinations, and unusual thought content. This total indicates a score of “very mild” on any two symptoms or at least mild on any one symptom (control samples typically score absent or “very mild” on these items [46]). “Moderate” psychosis will correspond to BPRS positive symptom subscale scores between 6 and 16, representing one standard deviation above the mean score for patients with psychotic major depression [47]. Additionally, at least one subscale item score must be > 4 to ensure that the psychosis symptom is associated functional impairment. Individuals with psychotic major depression typically have BPRS positive symptom subscale scores between 7 and 11, and a score of 6 best differentiates psychotic MDD from nonpsychotic MDD [47].

Participants who demonstrate any of the following criteria will be excluded from participation:

- Pregnancy or sensitivities to ingredients in the Climara® patch or Prometrium®, or their generics
- Bilateral oophorectomy
- Body mass index (BMI) extremes (<18 or >45 kg/m^2^) due to its cardiovascular risk
- PET-MR contraindications
- Use of psychotropic, hormonal preparations, or medication with unknown mood effects
- No axis I disorder history during the 2 years prior to perimenopause onset
- History of mood episodes requiring hospitalization
- Current mania
- History of suicide attempts within the past year or active suicidal ideation with intent or plan
- Neurological conditions (e.g., seizure/traumatic brain injury/stroke) or brain stimulation in past 6 months
- Endometriosis, undiagnosed ovary enlargement or vaginal bleeding, liver disease, breast cancer, cardiovascular disease, history of blood clots in legs or lungs, porphyria, type I or II diabetes mellitus, malignant melanoma, gallbladder or pancreatic or kidney disease, cigarette smoking, recurrent migraines with aura
- First-degree relative with premenopausal breast cancer, breast cancer in both breasts, or multiple relatives (>3) with breast cancer
- Poor cardiovascular health as determined by review of medical records and symptoms to rule out chronic, uncontrolled hypertension, history/presence of any heart conditions, dyspnea with or without exertion, orthopnea, chest pain/pressure, sense of rapid or irregular heartbeat/palpitations, exercise intolerance (i.e., METS <4), paroxysmal nocturnal dyspnea, unexplained syncope, unexplained lower extremity edema
- Current substance use disorder or history of moderate-to-severe substance use disorder within the past 2 years

### Who will take informed consent {26a}

All participants provide informed consent with the study coordinator prior to participating.

### Additional consent provisions for collection and use of participant data and biological specimens in ancillary studies {26b}

This trial does not involve collecting biological specimens.

### Interventions

#### Explanation for the choice of comparators {6b}

To evaluate the effects of estradiol while taking into account possible placebo effects, estradiol treatment is being compared to placebo treatment.

#### Intervention description {11a}

Participants will be randomized to either a 3-week estradiol (100 μg/day, via Climara® patch, or its generic) or placebo treatment. Four weekly clinical assessments will be conducted (baseline, week one, week two, and at the end of treatment) to assess changes in anhedonia and psychosis symptoms as well as blood serum levels of estradiol. Furthermore, participants will complete PET-MR scans at baseline and the end of treatment to examine neural and molecular responses to rewards.

#### Treatment arms

The estradiol treatment group will receive 100 μg/day of estradiol via the Climara® patch (or its generic) for 3 weeks, with weekly patch placement by the study coordinator. The same procedures will be used for the administration of the placebo patch. Upon completion of the 3-week protocol and post-treatment outcome measurement, participants in the estradiol group will receive 1 week of micronized progesterone at 200 mg/day, administered via the Prometrium® oral capsules (or its generic) combined with an additional week of estradiol (0.1mg/day, Climara® patch, or its generic). Study drugs and placebo patches will be maintained in the UNC Pharmacy Storeroom and dispensed by the UNC IDS to the study coordinator following the participant’s consent and eligibility assessment.

#### Dose justification

The Climara® patch is an FDA-approved treatment for menopausal and postmenopausal women experiencing vasomotor and genitourinary symptoms, with dosage ranging from 25 to 100 μg/day [48]. While estradiol treatment is not currently FDA-approved for treatment of perimenopausal affective disorders, there is empirical support for the efficacy at doses ranging from 100mcg/day [49] to 0.5mg/day [50]. Furthermore, to minimize adverse side effects, a biologically relevant dose will be administered for a short window. While adverse effects may be associated with longer duration usage in older, postmenopausal women [51], the current protocol is expected to pose minimal risk to participants [8].

#### Criteria for discontinuing or modifying the allocated intervention {11b}

Participants are free to withdraw from the study at any time, and this right will be reviewed during the consenting process. Investigators may discontinue study participation prior to completion of treatment for the following reasons: (1) intervention non-compliance; (2) the participant is lost-to-follow-up; (3) a medical condition or situation arises where continued participation is contraindicated; (4) a condition develops or an event occurs that meets exclusion criteria; (5) suicidal thoughts, plans, or intent is expressed; (6) or there are any other concerns about continued participation. In the case of severe mood symptoms or suicidal thoughts, the first three items on the Colombia-Suicide Severity Rating Scale will be administered by a member of the research team. If the participant expresses active suicidal ideation within the past week or expresses concerns about their ability to continue in the study, they will be evaluated by a licensed clinical psychologist or psychiatrist and will be discontinued from the study protocol. If symptoms are deemed otherwise unmanageable, inpatient admission to the university hospital’s psychiatry department will be arranged.

#### Safety assessments

A baseline medical health assessment is conducted prior to clearing participants for treatment. This includes blood panels and a pelvic exam/PAP test done by the study gynecologist or the participant’s personal provider if they choose. If the participant has had a pelvic exam/PAP test completed within 3 years prior to enrollment, a review of medical records can take the place of an updated exam. Participants must show no evidence of exclusionary conditions and have no history of abnormal labs or resolved abnormal labs as verified by additional testing and treatment. Furthermore, prior to each PET-MR visit, participants will complete a pregnancy test and MRI screening form to ensure there are no contraindications to scanning.

#### Strategies to improve adherence to interventions {11c}

Participants are instructed to contact the study team immediately if their patch begins falling off so that a new, blinded patch can be placed.

#### Relevant concomitant care permitted or prohibited during the trial {11d}

To be eligible to complete the study, participants must not be taking any psychotropic medication or supplement that is known to alter the mood. Study participants who elect to begin taking such medications or supplements during the 3-week treatment window will be discontinued from the study.

#### Provisions for post-trial care {30}

No harm is anticipated so there are no provisions prepared.

### Outcomes {12}

#### Mechanistic outcomes

The primary mechanistic endpoint in this trial will be neural and molecular response to rewards using simultaneous PET-MR, which will be measured at baseline and at the end of treatment. Scans will be collected on a Siemens Biograph mMR scanner. A whole-brain, T1-weighted MPRAGE image will be collected (1mm isotropic, TR=2400ms, TE=2.13ms). A whole-brain T2*-weighted BOLD sequence will be used for the acquisition of fMRI task data (3mm isotropic, TR=3000 ms, TE=30ms). Simultaneous acquisition of PET data will occur using [^11^C]raclopride PET imaging to estimate D2 and D3 receptor occupancy at baseline and during reward conditions. A bolus plus infusion protocol will be used with the following parameters: K_bol_=70 min, total radioactivity=15 mCi, mass dose=10 μg (specific radioactivity at time of bolus > 0.4 Ci/μmol). Listmode 3D emission data will be acquired from bolus administration through scan duration. The study will also acquire Siemens proprietary MR attenuation maps optimized for head imaging with a bone compartment to be used for attenuation correction.

This study uses a version of the monetary incentive delay (MID) task (for a review, see [52]), modified for PET-MR acquisition [53]. The task consists of a neutral block during which no monetary rewards are delivered, followed by reward blocks where varying magnitudes of rewards are delivered. Based on each participants’ reaction time performance, the task is programmed to code approximately 75% of responses as successful. Task trials consist of (1) the presentation of a blue polygon cue, followed by (2) the presentation of a green circle target stimulus cueing the participant to respond with a speeded button press, and then (3) the presentation of an outcome. For the neutral block, no monetary outcomes are delivered; rather, a gray rectangle is presented indicating a successful trial with no reward. Other outcomes include trial performance feedback (e.g., “Too Fast!” “Too Slow!” or “No Response!”). For reward blocks, task trials use the same structure, with a few minor differences. Instead of a stable blue polygon cue, different polygon cues are presented (e.g., square, triangle, pentagon, hexagon), which are associated with specific reward outcomes (e.g., no reward, 50 cents, one dollar, five dollars). Polygon cue-reward associations remain stable across trials. Thus, reward blocks capitalize on associative learning to optimize dopamine response to rewards [54]. Furthermore, compared to other versions of the MID task, a higher percentage of reward trials (75%) deliver reward outcomes, and many large rewards ($5) are offered, to increase reward anticipation response [55]. Consistent with the neutral block, for unsuccessful trials, performance feedback is delivered (e.g., “Too Fast!” “Too Slow!” or “No Response!”) in lieu of reward outcomes (e.g., no reward, 50 cents, one dollar, five dollars). The reward block begins approximately 40 min after [^11^C]raclopride bolus injection to allow for target/reference stabilization. This 40-min baseline acquisition period is used to estimate baseline binding potential.

For analysis of fMRI reward-related activation, functional data will be preprocessed, including motion correction, slice timing correction, normalization, and spatial smoothing. Event onsets will be convolved with a double-γ hemodynamic response function to model task-related activation. The primary task contrasts of interest will probe activation in reward versus neutral trials modeled separately during anticipation and outcome phases of the task. An a priori region-of-interest approach will be used in group-level analyses. Exploratory whole-brain analyses will also be conducted. All analyses will use a cluster-forming threshold of *p*<.001 and a cluster-corrected threshold of *p*<.05. With respect to molecular response to rewards estimated via PET, 1-min framewise post-scan reconstruction will be conducted. Motion correction will be applied using mutual information registration with a mid-frame reference. Binding potential will be estimated as the ratio of selectively bound ligand to non-displaceable ligand at tissue equilibrium. A dynamic occupancy model will be used to assess changes in binding potential secondary to dopamine release. Baseline binding potential (BP_ND_) and task-related changes in BP_ND_ (ΔBP_ND_) will be calculated using time-activity curves for regions of interest via a simplified reference tissue model (e.g., 3-parameter, single-exponential fit to the time activity curve). Non-vermis cerebellar regions will be used as the reference tissue.

Primary measures of neural activation will be estimated using mean percent signal change for the reward versus neutral contrast during anticipation. The primary molecular measures estimated via PET will include the average baseline dopamine binding potential and task-related changes in binding potential. For both neural and molecular measures, regions of interest will include the nucleus accumbens, caudate nucleus, and putamen. In addition, we will conduct exploratory fMRI analyses of activation in medial prefrontal regions during reward outcome processing.

#### Clinical outcomes

Clinical outcomes will include collecting measures of anhedonia (the SHAPS) and psychosis (the BPRS) symptoms.

### Participant timeline {13}

**Fig. 1:**
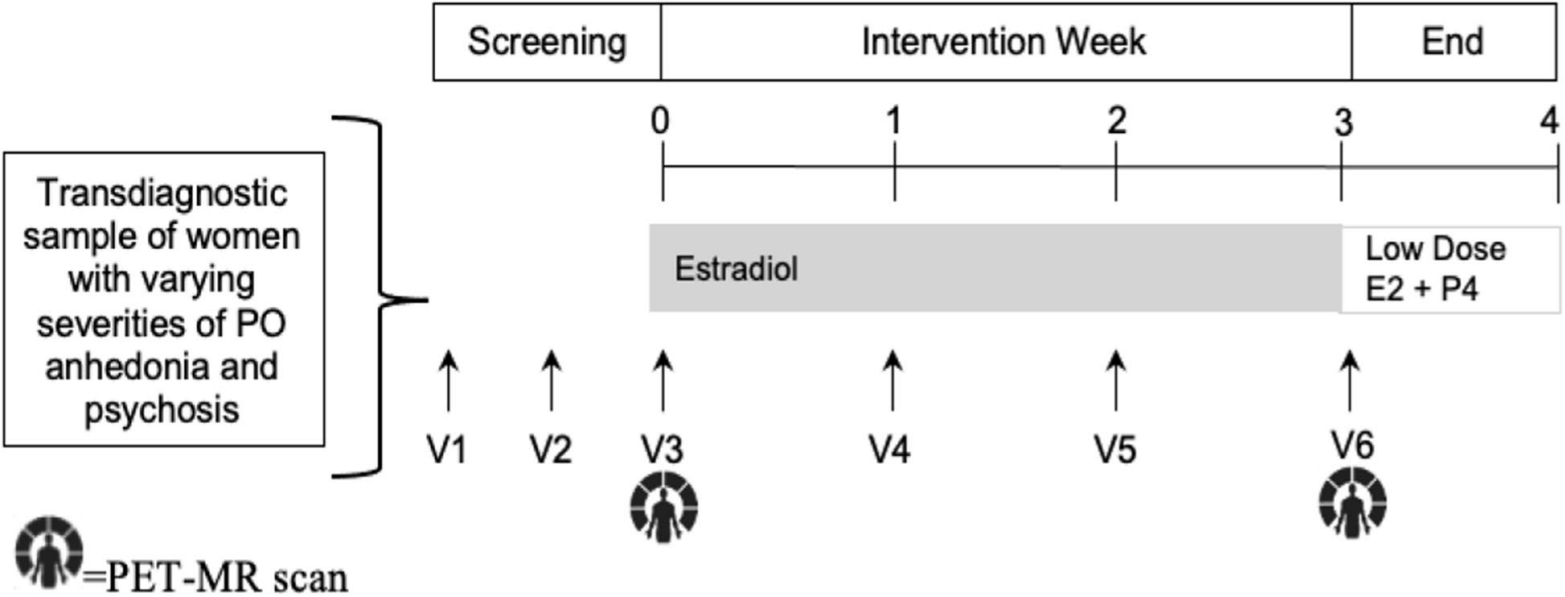
Study timeline

Figure 1 depicts the study timeline.

### Sample size {14}

For the first study objective, correlation analyses will be used to examine associations between baseline clinical measures (anhedonia and psychosis) and neural markers (striatal BOLD activation and baseline/reward-related dopamine release). Power analysis suggests that a sample of 85 participants will yield approximately 80% power to detect small-to-medium effects (smallest detectible *r*=.27) at *α*=.05, which is consistent with our in-house pilot data. With respect to the study’s second objective, longitudinal mixed models will be used to examine the effects of estradiol versus placebo on (1) symptom trajectories measured across four timepoints and (2) neural/molecular markers across two timepoints. A sample of 85 participants will yield 80% power to detect small-to-medium effects, with the smallest detectible effect *f*^2^=.05 for clinical outcomes and *f*^2^=.07 for brain changes. To determine associations between changes in symptoms and changes in neural/molecular reward response in the estradiol treatment group, intercorrelations will be examined in primary analyses. A sample of 43 participants will yield 80% power to detect medium-to-large effects, with the smallest detectible effect size of *r*=.41.

### Recruitment {15}

**Fig. 2:**
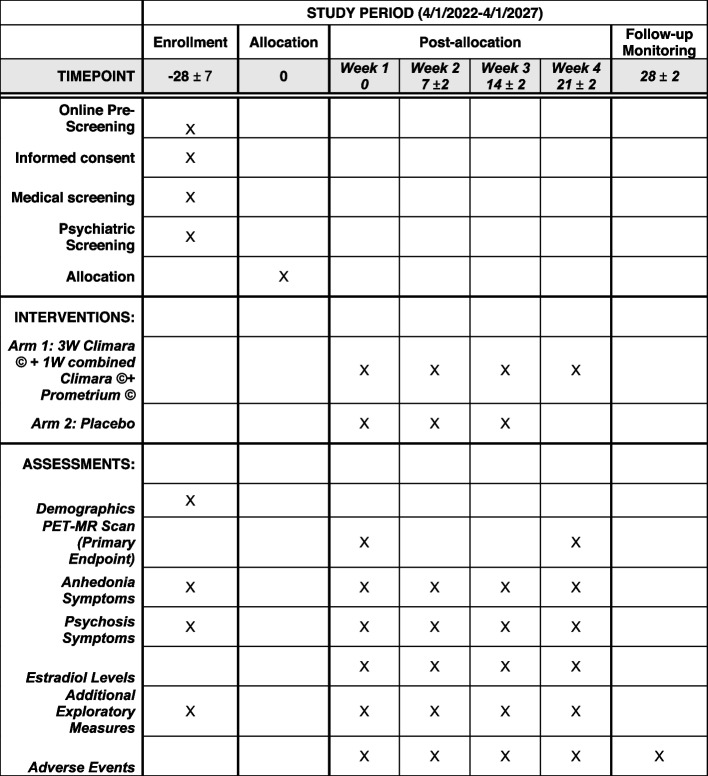
Schedule of enrollment, interventions, and assessments

Study advertisements will be posted on local and digital media (e.g., Facebook, Instagram, Twitter). Participants will also be recruited from the UNC Hospital Carolina Data Warehouse for Health, which is a repository used for research participant recruitment in which patients of the UNC Health Care System can voluntarily enroll. Study flyers will also be posted at UNC clinical service units, including gynecology, primary care, psychiatry, psychology, and women’s health (Fig. 2).

## Assignment of interventions: allocation

### Sequence generation {16a}

Computer-generated randomization allocated participants to either active or placebo treatment condition without stratification.

### Concealment mechanism {16b}

After the determination of eligibility, the IDS will be notified to randomize the participant to either the estradiol (100 μg/day Climara® patch, or generic) or placebo (hydrocolloid patch) condition. Unblinded study medication will be dispensed to the study coordinator, who will blind and administer the medication to participants. In accordance with the study’s double-blind protocol, (1) all study personnel involved in the design, clinical outcome data collection, and analysis and (2) the research participant will be blind to treatment group assignment. The placebo and treatment patches will not contain any distinguishing information or feature visible to the research participant.

### Implementation {16c}

The study randomization list will be generated by an independent biostatistician and retained by the UNC Investigational Drug Service (IDS).

## Assignment of interventions: blinding

### Who will be blinded {17a}

For study personnel, the blind will be maintained by the UNC IDS until the end of the study, except for the study coordinator responsible for blinding and administering treatment.

### Procedure for unblinding if needed {17b}

Participants will be unblinded at the end of treatment/outcome data collection, at which point active treatment participants will commence 1 week of progesterone (200 mg/day, Prometrium® oral capsules, or its generic) combined with an additional week of estradiol (0.1mg/day, Climara® patch, or its generic) to stimulate menstruation and reduce the risk of endometrial hyperplasia.

## Data collection and management

### Plans for assessment and collection of outcomes {18a}

The primary clinical outcomes measures will examine anhedonia and psychosis-spectrum symptoms at treatment baseline, week one, week two, and the end of treatment (week 3). The Snaith–Hamilton Pleasure Scale (SHAPS) [38], a 14-item self-report questionnaire assessing hedonic behaviors with a score range of 14–56, will be used as a dimensional measure of anhedonia. The Brief Psychiatric Rating Scale, an 18-item, rater-administered scale measuring psychotic symptoms and anti-psychotic treatment response, will be used as a dimensional measure of psychosis [39, 40]. A psychosis subscale score will be calculated for primary analyses, consisting of scores from four items: conceptual disorganization, suspiciousness, hallucinations, and unusual thought content.

Additional measures will be collected to assess psychological and physiologic symptoms associated with the perimenopausal transition. Measures include the following:

- Inventory of Depression and Anxiety Symptoms (IDAS-II [56]), a 64-item self-report measure with good psychometric properties that has been validated for use with reproductive mood disorder samples
- Clinical Global Impression (CGI) scale [57]: assessment of functional impairment to be collected at pre-treatment, week one, week two, and post-treatment
- Life Events Survey [58]: survey of stressful life events that may moderate effects of mood symptoms [59]
- Green Climacteric Scale [60]: 21-item gold-standard self-report measure for assessment of climacteric symptoms (vasomotor, somatic, anxiety, depression)
- Pittsburgh Sleep Quality Index (PSQI [61]): 9-item self-report questionnaire measuring sleep quality/patterns in older adults
- DSM-5 Self-Rated Level 1 Cross-Cutting Symptom Measure—Adult [62]: self-report questionnaire assessing symptoms relevant across different mental health diagnoses
- The 7-item anxiety scale (GAD-7) [63]: brief self-report anxiety scale
- Patient Health Questionnaire (PHQ)-9 [64]: brief 9-item questionnaire to assess depression severity
- World Health Organization Disability Assessment Schedule 2.0 [65]: self-report assessment of functioning across six domains (cognition, mobility, self-care, social interaction, life activities, participation)

These measures will be used in secondary and/or sensitivity analyses to examine the effect of covariates (in particular, climacteric symptoms, sleep quality, stressful life events).

### Plans to promote participant retention and complete follow-up {18b}

Participants will have the opportunity to receive up to $500 in total compensation for completion of study procedures. They will receive $60 for their first screening visit (completion of the SCID-5 and symptom questionnaires) and $30 for the second screening visit (blood panel and completion of a gynecological exam or medical records from an exam in the past three years). For the baseline and post-treatment PET-MR imaging sessions, participants can earn between $140 and $200 per session depending on their performance on the MID task. A variable compensation rate is necessary for these neuroimaging sessions to examine neural and molecular biomarkers related to motivation to win money. Furthermore, during weeks one and two of treatment, they will earn $55 per visit (brief interview, questionnaires to assess anhedonia/psychosis, and a blood draw). In the case of withdrawal from the study, a prorated payment schedule will be applied, including an hourly rate for PET-MR visits of $50 per hour and up to $150 total. Study coordinators are available to conduct clinical assessments at the home of participants or over zoom to increase retention and collect all clinical data.

### Compliance monitoring

Treatment compliance will be assessed via participant self-report. Blood samples will also be collected on a weekly basis as a biological measure of compliance. Samples will be used to measure serum estradiol levels using the enzyme-linked to immunosorbent assay (ELISA). In the event the study treatment protocol is not followed, the participant’s data will be excluded from analyses.

### Data management {19}

A CONSORT diagram tracking log will be used to track the number of participants that were pre-screened, consented, cleared for treatment, and completed the study, as well as withdrawals. Case report forms containing hard copies of data will be stored within a locked filing cabinet in a secure, locked room. Data from case report forms, including clinical measures and laboratory data, will be entered digitally and stored on HIPAA-compliant servers. REDCap will be used for secure collection and storage of digital survey and questionnaire data. Upon completion of the clinical trial, study documents will be retained for a minimum of seven years.

### Confidentiality {27}

Participant identification codes are assigned at the time of screening. PIs and study coordinators have access to personal identifiers of enrolled participants and medical records through their dual roles as researchers and care providers.

### Plans for collection, laboratory evaluation, and storage of biological specimens for genetic or molecular analysis in this trial/future use {33}

No biological specimens were collected in this trial.

### Case report form

For each consented participant, a case report form will be generated that summarizes screening and study data, including deidentified hard copies of clinical measures. Participants will be identified by their alphanumeric identifier on this form to maintain confidentiality. Upon completion of the study, case report forms will be maintained for a minimum of seven years.

## Statistical methods

### Statistical methods for primary and secondary outcomes {20a}

The first objective of this study is to examine baseline (i.e., pre-treatment) associations between anhedonia and psychosis and neural and molecular responses to rewards. Associations between clinical measures and mechanistic outcomes will be estimated via correlation analysis. The second objective is to evaluate the experimental effects of estradiol versus placebo on clinical outcome measures and mechanistic outcomes. Mixed effects models will be used to examine the effects of time, treatment group, and their interaction on clinical outcomes (four timepoints, random intercept and slope) and neural and molecular outcomes (two timepoints, random intercept only), with age as a covariate. A separate model will be generated for each clinical outcome measure (anhedonia and psychosis) and each neural and molecular outcome (activation during reward anticipation and BP_ND_ and task-related ΔBP_ND_ for striatal regions of interest). Exploratory whole-brain analyses will also be conducted for fMRI activation during reward anticipation. Within the estradiol treatment group only, we will examine how biomarkers of reward response predict changes in clinical outcome measures. We will examine intercorrelations between anhedonia or psychosis and neural and molecular reward response, using residualized gain scores to quantify changes in clinical measures. We will also use mixed effect models to examine how changes in neural and molecular reward response predict variability in clinical outcome trajectories, with a separate model for each clinical outcome and each neural and molecular change score. Maximum likelihood estimation will be used in all mixed effect models to accommodate missing data (e.g., SAS proc mixed). When multiple comparisons are conducted to test a hypothesis, appropriate correction will be applied to achieve a corrected *p*-value of 0.05. For clinical outcome hypotheses, both a per-protocol and an intent-to-treat analytic approach (e.g., utilizing all available data, even for participants who did not finish treatment to completion) will be used. For mechanistic hypotheses, a per-protocol approach will be used, including only participants who completed the baseline PET-MR scan, 3 weeks of estradiol treatment, and the post-treatment PET-MR scan.

### Methods for additional analyses (e.g., subgroup analyses) {20b}

No subgroup analyses on the outcomes are planned, though suspension or premature discontinuation and closure of the study may occur if the participation in study procedures poses unexpected, significant, or unacceptable risk, as described in section {22} addressing adverse events.

### Interim analyses {21b}

No interim analyses on the outcomes are planned.

### Methods in analysis to handle protocol non-adherence and any statistical methods to handle missing data {20c}

We will report any differences in consent, withdrawal, and loss-to-follow-up by randomization arm. We do not have plans for imputation for missing data.

### Plans to give access to the full protocol, participant-level data, and statistical code {31c}

Access to a cleaned, deidentified dataset and code may be requested after the study is completed and accompanying manuscripts are published. The primary aim is a study-wide analysis and will be disseminated as such. Any publications and presentations prior to the release of the primary results will not impede the integrity of those results.

## Oversight and monitoring

### Composition of the coordinating center and trial steering committee {5d}

The principal investigators will serve as study monitors. They will ensure compliance with the investigational plan as well as IRB regulations, hosting weekly study team meetings focusing on progress monitoring and safeguarding against unanticipated problems. Protocol deviations will be discussed in weekly review meetings and corrective actions will be identified. Furthermore, the principal investigators will be notified of serious adverse events within 24 h and non-serious adverse events within 7 days and follow-up to resolution.

Quality assurance measures will also be implemented to ensure protocol compliance and data quality. During training of study personnel, detailed review and feedback will be provided regarding informed consent procedures. Reliability checks will be implemented on a routine basis for the administration of clinical outcome measures, with a minimum of 20% reviewed. Research assistants will be trained to enter and review data quality in REDCap. Data from REDCap will be downloaded regularly as an excel file to a secure drive and inspected for accuracy and completeness. Furthermore, a representative sample of source data from the case report form will be checked against the REDCap study database to ensure accurate entry. A data manager will also review REDCap data for plausibility and completeness and will flag and follow up on potential errors.

### Composition of the data monitoring committee, its role, and reporting structure {21a}

Independent oversight of study safety will be conducted by the Data Safety Monitoring Board (DSMB). The DSMB is responsible for monitoring and minimizing study risks, reviewing modifications to risk management protocols, monitoring enrollment progress, and recommending study continuation based on ongoing risk versus benefit analysis. The DSMB will receive biannual reports summarizing safety and enrollment data as well as any revisions to the study protocol. The DSMB will also be notified within 24 h of any serious adverse events.

### Adverse events reporting and harms {22}

Adverse events will be monitored by study staff from the treatment baseline through the final study visit. Participants will be advised during the consenting process to notify study staff immediately if they experience any new or unusual side effects during participation. Upon initiation of treatment, weekly check-ins will be completed to inquire about potential adverse events. Non-leading questions will be asked (e.g., “How have things been going for you? How has your mood been? How have you felt physically? Have you noticed anything different in the past week?”). Participants will be provided weekly handouts on potential adverse side effects associated with estradiol treatment. Serious adverse events, as defined by the FDA, will be reported to the principal investigators and the Data Safety Monitoring Board (DSMB) within 24 h, to the university IRB within 5 days of occurrence, and, for unexpected serious adverse events, to the study sponsor within ten business days. Other adverse events that are non-serious, but deemed study related, will be reported biannually to the DSMB.

Ongoing trial safety monitoring, compliance, and progress will be conducted by the principal investigators, the DSMB, and the sponsor. The DSMB will convene biannually, or sooner if required (e.g., in the case of serious adverse events), to formally evaluate the progress and safety of the trial. The following circumstances may warrant suspension or premature discontinuation and closure of the study:

- Participation in study procedures poses unexpected, significant, or unacceptable risk (e.g., three or more serious adverse events reported).
- There are findings of (a) insufficient compliance with the study protocol, (b) data are not sufficiently complete or evaluable, or (c) trial progress is determined insufficient.
- There has been a clear demonstration of trial (a) efficacy or (b) futility.

In the case of premature termination or suspension, the principal investigators will inform the study participants, the IRB, and the study sponsor, citing the specific reason(s) for the suspension/termination decision. In the case of suspension, the study may resume when concerns are determined to be adequately addressed.

### Frequency and plans for auditing trial conduct {23}

Internal audits of this trial are conducted quarterly. Intervention implementation and quality improvement are addressed at weekly team meetings with the study PIs, co-investigators, and study staff.

### Plans for communicating important protocol amendments to relevant parties (e.g., trial participants, ethical committees) {25}

Changes to the study protocol must be approved by the IRB and reported to the study sponsor. Furthermore, any modifications to risk management protocols will be approved by the DSMB. If significant deviations from the existing study protocol occur, they will be reported to the IRB within 2 weeks as well as in annual progress reports to the sponsor and to the DSMB during their biannual meeting schedule, or within 4 weeks for serious violations (e.g., confidentiality breach). We used the SPIRIT Checklist to assist with presenting this protocol.

## Dissemination plans {31a}

In accordance with NIH sponsor policy, the results and accomplishments of study activities will be made publicly available. All peer-reviewed manuscripts will be submitted to the public-access PubMed Central digital archive upon acceptance for publication. Every attempt will be made to publish the clinical trial results in peer-reviewed journals. Furthermore, upon completion of the clinical trial, study data will be deidentified and the archived data will be shared publicly on the NIMH Data Archive (NDA). In accordance with the NIH policy on the Dissemination of NIH-Funded Clinical Trial Information and the Clinical Trials Registration and Results Information Submission rule, the clinical trial will be registered at ClinicalTrials.gov and results of the study will be submitted to this website.

## Discussion

Affective and psychotic conditions show increased prevalence during the perimenopause compared to pre- and postmenopausal periods [3, 4]. While fluctuations in ovarian hormones, in particular estradiol, are thought to trigger the onset of perimenopausal affective [7] or psychotic symptoms [5], not all women show susceptibility. Furthermore, estrogen treatment shows empirical support for reducing perimenopausal affective [8–10] and psychosis symptoms [5]; however, benefits are not universal. Characterizing neuromolecular pathways by which estrogen fluctuations during the perimenopausal period (e.g., via ovarian quiescence or estrogen treatment) trigger affective and psychotic symptoms is warranted. The mesolimbic dopamine pathway shows dysfunction in both depression [20, 21] and psychosis [24, 25]. Furthermore, fluctuations in estradiol have been found to alter the function in this pathway [26]. Preclinical evidence suggests the mesolimbic dopamine pathway may mediate the relationship between ovarian hormone withdrawal and depressive symptoms [12]. The PEEPS study will be the first mechanistic clinical trial to probe the relationship between neural and molecular measures of reward system function and estrogen-mediated changes in affective and psychosis behaviors during the perimenopausal period. The results of this trial will inform future studies predicting susceptibility to perimenopausal affective and psychotic conditions and developing targeted treatments.

In accordance with the NIMH RDoC framework, this trial aims to characterize the etiology of psychiatric symptoms experienced during the perimenopausal period in terms of varying degrees of impairment in psychological and biological systems linked to Positive Valence System dysfunction [66]. Specifically, this mechanistic trial will use transdiagnostic inclusion criteria measuring psychiatric symptom dimensions (i.e., anhedonia, psychosis) linked to both the perimenopausal transition and dysfunction in brain reward system functioning. In complement and with the intent of increasing sample homogeneity, the trial will use DSM-based criteria to exclude participants who present with conditions linked to reward system dysfunction with non-endocrine-related etiology. By applying this integrated RDoC framework with DSM-based exclusion criteria in a pharmacologic, simultaneous PET-fMRI design, this study may serve as a framework for probing the neuromolecular basis by which specific pharmacologic compounds impact broad psychological domains implicated in mental health and well-being. In the context of reproductive transitions and associated mental health symptoms, this paradigm could be modified to address the effects of various sex hormones on neurotransmitter systems to characterize susceptibility and evaluate treatments. Such research has the potential to broadly enhance scientific understanding of relationships between sex hormones, neurotransmitter system function, and psychiatric symptom dimensions in women across hormone transitions, including puberty, menstruation, pregnancy, and menopause.

## Trial status

This trial was granted UNC-Chapel Hill Institutional Review Board approval on 12/16/2021 and approval by its Data Safety Monitoring Board in April 2022. Recruitment was initiated on 04/01/2022. This is version 1.6 of the study protocol (06/09/2022). Recruitment will end December 31, 2026.

## Data Availability

Upon completion of the study, the data will be made available for download from the NIMH National Database Archive (NDA), collection #4215.

## Abbreviations

PEEPs: Perimenopausal Effects of Estradiol on Anhedonia and Psychosis Study
BPRS: Brief Psychiatric Rating Scale
RDoC: Research Domain Criteria
BOLD: Blood oxygen level dependent
MID: Monetary incentive delay
SCID-5: Structured Clinical Interview for the DSM-5
STRAW: Stages of Reproductive Aging Workshop
SHAPS: Snaith–Hamilton Pleasure Scale
BMI: Body mass index
